# Perturbation-Induced Protective Arm Responses: Effect of Age, Perturbation-Intensity, and Relationship with Stepping Stability: A Pilot Study

**DOI:** 10.3390/brainsci12070953

**Published:** 2022-07-20

**Authors:** Woohyoung Jeon, Shuaijie Wang, Tanvi Bhatt, Kelly P. Westlake

**Affiliations:** 1 Department of Physical Therapy and Rehabilitation Science, University of Maryland School of Medicine, Baltimore, MD 21201, USA; wjeon@som.umaryland.edu; 2 Department of Physical Therapy, University of Illinois at Chicago, Chicago, IL 60612, USA; sjwang4@uic.edu

**Keywords:** falls, balance, aging

## Abstract

During balance recovery from slip perturbations, forward flexion (elevation) of the arms serves to counterbalance the posteriorly displaced center of mass (CoM). We aimed to investigate whether aging affects modulation of arm responses to various intensities of unpredictable slip perturbations and whether arm responses are related to compensatory stepping stability. Ten healthy young adults and ten healthy older adults participated. Participants were asked to react naturally to three randomly administered levels of slip-like surface perturbations (intensity 1 (7.75 m/s^2^), intensity 2 (12.00 m/s^2^) and intensity 3 (16.75 m/s^2^), which occurred by means of forward acceleration of the treadmill belt while standing. Kinematic data were collected using a motion capture system. Outcomes included arm elevation displacement, velocity, and margin of stability (MoS) of compensatory stepping. The results reveal no modulation of arm elevation velocity in older adults from perturbation intensity 1 to 2, whereas younger adults demonstrated progressive increases from intensity 1 to 2 to 3. At intensity 3, older adults demonstrated reduced maximal arm elevation velocity compared to younger adults (*p* = 0.02). The results in both groups combined reveal a positive correlation between maximal arm elevation velocity and first compensatory step MoS at intensity 3 (*p* = 0.01). Together, these findings indicate age-related decreases in arm response modulation and the association of arm elevation response with protective stepping stability, suggesting that fall prevention interventions may benefit from an emphasis on arm elevation velocity control in response to greater perturbation intensities.

## 1. Introduction

Each year, one out of four community-dwelling older adults fall and the rate of fall related injuries leading to loss of function and independence increases with age [1,2]. Falls are the leading cause of fatal and non-fatal injuries and the leading cause traumatic brain injury (TBI) in older adults. In addition to TBI and fractures, nonfatal falls frequently lead to reduced levels of activity, fear of falling, and reduced quality of life [3,4]. In 2015, it was estimated that about $50 billion was spent on medical costs related to fatal and non-falls [5]. As a result of these substantial personal and financial burdens, falls in older adults are a major public health concern. Therefore, increasing the understanding of normal and im-paired responses to balance perturbations that are known to lead to falls such as slips and trips is critical to identify balance rehabilitation training targets for fall prevention.

Reactive balance recovery strategies following an unexpected loss of balance are crucial to the prevention of head trauma and other major injuries. While the focus has predominantly been on understanding lower limb stepping responses or arm responses to feet-in-place perturbations, there is increasing evidence that responses of the arms are critical to balance recovery. For example, the lack of arm response following slip perturbations have been found to restrict control of whole body angular momentum in all the directions during balance recovery [6,7]. On the flip side, the arm response immediately after trip perturbations improves control of trunk axial rotation for balance recovery. More specifically, a prolonged swing of both arm toward the tripped side in the transverse plane contributes to greater trunk rotation further to the non-tripped side, thereby allowing the increased length of compensatory stepping during balance recovery [8].

Arm movements following balance and gait disturbances act in a coordinated manner with lower extremity responses despite not being directly stretched by the perturbation [9,10]. The onset of arm movements detected by EMG activation of the deltoid muscle has even been shown to occur prior to perturbed leg muscle activation in response to an unexpected slip [11], suggesting a time-critical initial role of arm response in adjusting the center of mass (CoM) to restore postural stability [12]. It is therefore essential that all aspects of a balance recovery response, including arm movement, are studied to understand the mechanisms of fall prevention more completely. In a counterbalancing role, arm flexion (elevation) helps to reverse the direction of a fall and adjust the CoM so that a protective step may be taken. For example, following a trip, increased upward linear momentum resulting from rapid arm elevation serves to reduce CoM forward momentum allowing a more effective recovery step to be taken [13]. Similarly, following a rapid forward slip, an arm elevation response is used to help shift the CoM forward, thereby decelerating posterior CoM displacement during a recovery step [6]. 

The ability to modulate balance responses enables one to effectively restore postural stability by matching responses to various fall characteristics, including velocity, displacement, and direction. To do so, the central nervous system relies on the integration of somatosensory, visual, and vestibular feedback and cortical motor responses to control and coordinate upper and lower limb responses [14,15]. Thus, age-related changes in the reliability of sensory input and slowed central processing may lead to an impaired ability to match balance responses to perturbation characteristics in older adults. In terms of the stepping response, older adults have been shown to modulate recovery step length to restore stability at lower, but not high perturbation intensities [16]. Modulation of the arm response can occur through a change in strategies or adjustment in movement velocity and displacement to counterbalance the fall. One example is to adjust from a counterbalancing motion such as stepping responses and trunk movement at lower intensities to a protective motion with arm response at higher intensities in order to cushion an impending fall that is perceived unavoidable. This switch in arm strategies was found in young adults in response to low and high slip perturbations, but not in older adults [17]. 

Although age-related differences have been identified in response strategies to varied perturbation intensities, age-related differences in the ability to modulate the magnitude of the arm response through effector control (e.g., muscle activation and kinematics) to restore postural stability has not yet been investigated. Therefore, we aimed to investigate age-related differences in the modulation of arm responses to different slip-like perturbation intensities as part of a whole-body stepping response. We hypothesized that older adults would demonstrate reduced ability to scale arm elevation amplitude and velocity to match various perturbation intensities compared to younger adults. 

## 2. Materials and Methods

A between-group cross-sectional design was used to compare kinematic differences of arm responses and balance stability following a slip between older and younger adults.

### 2.1. Participants

Ten older healthy adults (age: 62.5 ± 4.65, M:7/F:3) and ten young healthy adults (age: 28.2 ± 4.76, M:4/F:6) participated in the study, and all participants were recruited using convenience sampling. To ensure independent functioning and intact cognition, participants with the ability to walk 8 m without any assistive device and a score of ≥26/30 on the Mini-Mental State Examination were included. In addition, all participants were screened using a general health questionnaire to ascertain their health status. Individuals were excluded if they reported any neurological (e.g., Parkinson’s disease, Alzheimer’s disease, stroke, visual and/or vestibular impairment), musculoskeletal, cardiopulmonary, or any other systemic disorders. Additionally, individuals with heel bone density (measured using Lunar Achilles Insight) T-score < −1.0, used to identify osteopenia/osteoporosis which may interfere with balance responses, were excluded. Lastly, individuals with the inability to understand English were excluded to ensure that they could follow all the instructions provided during their participation.

The study was conducted according to the guidelines of the Declaration of Helsinki, and approved by the Institutional Review Board of the University of Illinois Chicago. Informed consent was obtained from all subjects involved in the study.

### 2.2. Procedures

**Figure 1:**
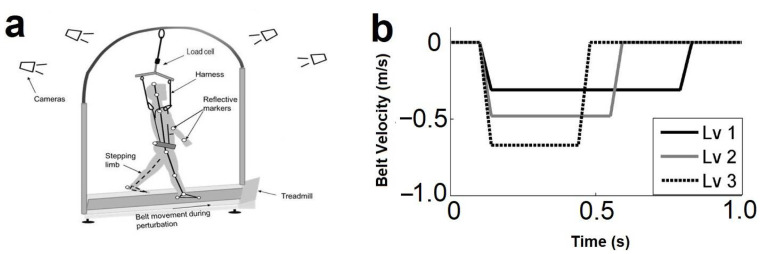
(**a**) Experimental set-up for slip-like perturbations. Reprinted with permission from Ref. [16]. 2016, Patel, P.J., (**b**) perturbation profiles of a treadmill slip. Each slip perturbation consists of three phases: acceleration, constant velocity, and deceleration. In the first phase (acceleration), the perturbation intensities were 7.75 m/s^2^ (intensity 1), 12.00 m/s^2^ (intensity 2), and 16.75 m/s^2^ (intensity 3). In the second phase (constant velocity), the perturbation intensities were 0.31 (intensity 1), 0.48 (intensity 2), 0.67 m/s (intensity 3). All the three perturbation intensities led to the same slip distance of 0.2 m.

Participants stood in a comfortable stance position on a specialized treadmill (ActiveStep Simbex, Lebanon, NH, USA). An overhead safety harness was worn to prevent participants’ knees from touching the treadmill belt in case of a slip-induced ‘fall’. Slip-like perturbations on the treadmill were induced by accelerating the belt in a forward direction from a static standing position within 2–4 s after the “start” sound. The first trial was a familiarization trial at intensity level 2 in which participants were instructed to react naturally to maintain their balance and prevent themselves from falling. Then, participants were exposed to one perturbation at each level (intensity 1, 2, 3). Each perturbation was presented 5–20 s after the participants resumed a comfortable stance. Perturbation trials were administered at three different intensities (defined by alterations in the acceleration of the treadmill belt): 7.75 m/s^2^ (level 1), 12.00 m/s^2^ (level 2), and 16.75 m/s^2^ (level 3) with a displacement of 0.20 m (see Figure 1). The order of presentation for the different intensity perturbations was randomized and blocked for each participant.

An eight-camera motion capture system with a sampling rate of 120 Hz was used to record full-body kinematics (Motion Analysis Corporation, Santa Rosa, CA, USA). Joint centers and center of mass (CoM) were determined using the Helen Hayes marker set with 29 markers placed on bilateral bony landmarks, head and trunk (Davis et al., 1991). The whole-body CoM was calculated as a weighted sum of the CoM of all the segments defined by the Helen Hayes markers set (the pelvis on the left and right anterior-superior iliac spine (ASIS), the sacrum (L4-L5), thigh and shank, lateral femoral condyle, lateral malleolus and second metatarsal head. Dempster’s normative anthropometric data were used to determine the limb segments. Kinematic marker data were low-pass filtered using a fourth-order Butterworth filter with a cut-off frequency of 6 Hz [18]. The kinematic variables were computed using custom-written algorithms in MATLAB version 2020b (The MathWorks Inc., Nactick, MA, USA).

The positional change of the elbow and heel markers in the vertical direction following a slip perturbation onset was used to determine the onset latency of arm elevation and first compensatory step lift off (LO). The elbow marker trajectory in the vertical direction was used to calculate maximal displacement and velocity of arm elevation from perturbation onset to first compensatory step touch down (TD). Elbow marker trajectory during the arm reaction was calculated from the elbow marker displacement value relative to the shoulder marker displacement in the vertical direction. This value was then normalized to the upper arm segment (the distance between the shoulder and elbow marker positions, the moment arm of the upper arm movement) as shown in the following equation:

Normalized elbow marker displacement (%) =

$$\frac{\left|Displacement\;of\;elbow\;marker-displacement\;of\;shoulder\;marker\right|\times 100}{Distance\;beteen\;the\;shoulder\;and\;elbow\;markers}$$

To measure compensatory stepping stability, the minimum distance from extrapolated CoM (x + v/ω_0_, where x and v are the CoM position and instantaneous CoM velocity, respectively, and ω_0_ is angular frequency, $\sqrt{g/l}$, *g* = gravity, *l* = the length between the CoM and foot) to the posterior boundary of the base of support (BoS) was quantified in the A–P direction [19]. The posterior boundary of the BoS was defined using heel marker position in the A–P direction. The onset timing of first compensatory step LO and TD was determined based on the positional change of heel marker in the vertical direction.

### 2.3. Statistical Analysis

The Shapiro–Wilk test was used to examine the normality of data. All variables met the assumption of normality. To investigate the presence of any main effects and interactions for onset latencies and kinematics (maximal arm elevation displacement, velocity, and compensatory step stability at TD), a two-way mixed ANOVA (within subject factor: perturbation intensity, between subject factor: age) was used, followed by Tukey’s post hoc tests for pairwise comparison when indicated. A paired samples t-Test was used to compare onset latency between arm and foot at each perturbation intensity. Pearson’s correlation (r) was used to examine the correlation between the maximal arm elevation velocity and compensatory step stability at first compensatory step TD across all perturbation intensities and at each perturbation intensity (intensity 1, 2, and 3). SPSS statistical software (IBM SPSS Statistics 25; Chicago, IL, USA) was used for all statistical analysis with an established a priori alpha level of 0.05.

## 3. Results

One younger subject’s data were excluded from statistical analysis because no arm reactions were used for balance recovery for any of the perturbation intensities.

### 3.1. Onset Latency of Arm Elevation and First Compensatory Step LO

There was no main effect of age (F = 0.035, *p* = 0.854) or intensity level (F = 1.827, *p* = 0.193) for arm elevation onset latency. There was also no main effect of age (F = 0.979, *p* = 0.336) or intensity level (F = 2.149, *p* = 0.149) for the onset latency of first compensatory step LO.

**Figure 2:**
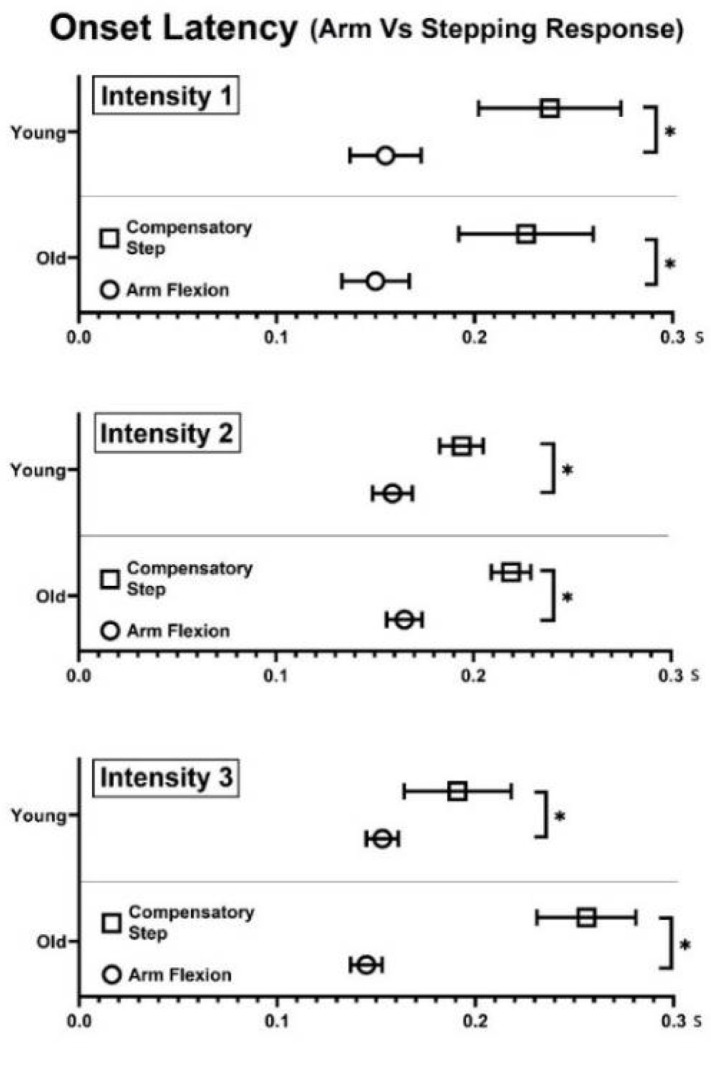
Onset latency of arm elevation and stepping response (first compensatory step lift-off) following a slip perturbation. Three different perturbation intensities (7.75 m/s^2^ (intensity 1), 12.00 m/s^2^ (intensity 2), and 16.75 m/s^2^ (intensity 3) were tested with a displacement of 0.20 m. * represents difference between arm and stepping responses, *p* < 0.05. Error bars display standard error.

When comparing the onset latency between arm elevation and the first compensatory step, arm elevation was found to occur earlier than first compensatory step LO at all three perturbation intensities in both age groups (all *p* < 0.05, see Figure 2).

### 3.2. Displacement and Velocity of Arm Responses

#### 3.2.1. Arm Elevation Displacement

**Figure 3:**
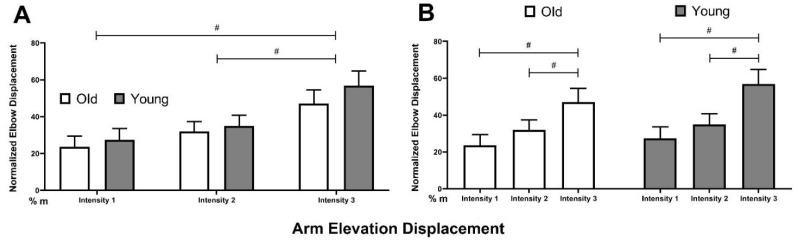
Maximal arm elevation displacement from perturbation onset to compensatory step touch down. # represents a difference between intensity levels, *p* < 0.05. Error bars display standard error. (**A**) Difference between two age groups at each perturbation intensity, (**B**) Difference between levels of perturbation intensity in each age group.

There was a main effect of perturbation intensity on maximal arm elevation displacement (F = 15.60, *p* < 0.01). Post hoc testing revealed that maximal arm elevation displacement at intensity 3 (51.96 ± 5.47% m) was greater than intensity 1 (25.44 ± 4.31% m, *p* < 0.01) and intensity 2 (33.45 ± 3.97% m, *p* < 0.01, Figure 3A). There was also no main effect of age on maximal arm elevation displacement (F = 0.54, *p* = 0.46, Figure 3B).

#### 3.2.2. Arm Elevation Velocity

**Figure 4:**
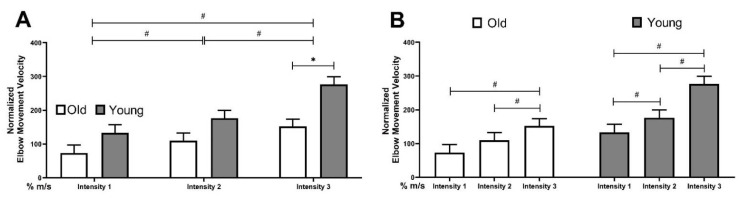
Maximal arm elevation velocity from perturbation onset to compensatory step touch down. # represents a difference between intensity levels, *p* < 0.05. * represents a difference between age groups, *p* < 0.05. Error bars display standard error. (**A**) Difference between two age groups at each perturbation intensity, (**B**) Difference between levels of perturbation intensity in each age group.

There was a main effect of perturbation intensity (F = 41.48, *p* < 0.01) and age (F = 7.82, *p* = 0.01) on maximal arm elevation velocity. Intensity 3 showed the highest velocity (214.88 ± 15.43% m/s, *p* < 0.01), followed by intensity 2 (143.03 ± 16.36% m/s, *p* < 0.01), and then intensity 1 (103.31 ± 16.67% m/s, *p* < 0.01, Figure 4A) had the lowest velocity. Compared to younger adults, the maximal arm elevation velocity was slower for older adults (old: 112.08 ± 20.50%m/s, young: 195.40 ± 21.63%m/s, *p* = 0.01). There was an interaction between perturbation intensity and age for the maximal arm elevation velocity (F = 4.73, *p* = 0.02). Older adults demonstrated significantly reduced arm elevation velocity at intensity 3 compared to younger adults (see Figure 4A). In addition, post hoc analysis revealed that maximal arm velocity was progressively increased from intensity 1 to 2 to 3 in younger adults (intensity 1 vs. 2: *p* = 0.03, intensity 2 vs. 3: *p* = 0.01), whereas older adults showed no difference between intensity 1 and 2 (*p* = 0.07, Figure 4B).

### 3.3. Relationship between Maximal Arm Elevation and Stepping Stability at TD

**Figure 5:**
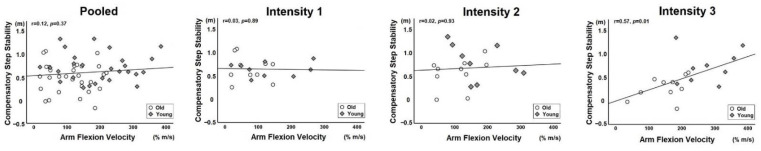
Correlation between maximal arm elevation velocity and first compensatory step stability at TD at each perturbation intensity. Pooled: combined set of all three perturbation intensities. There was a positive correlation at intensity 3.

There was no correlation between maximal arm elevation velocity and first compensatory step stability at TD in the pooled data (perturbation intensities and age groups combined, (r = 0.12, *p* = 0.37). When intensity levels were assessed individually, a positive correlation was observed at intensity 3 (r = 0.57, *p* = 0.01), whereas no correlation was found at intensity 1 (r = 0.03, *p* = 0.89) and intensity 2 (r = 0.02, *p* = 0.93, see Figure 5).

## 4. Discussion

The purpose of this study was to investigate age-related differences in the ability to modulate arm responses induced by different intensities of slip-like perturbations and the relationship of arm responses with compensatory stepping stability. The main finding was reduced maximal arm elevation velocity in older adults at the highest intensity (level 3). The positive correlation found between maximal arm elevation velocity and first compensatory step stability at TD at level 3 suggests that age-related diminished arm response for balance control is associated with increased instability during balance recovery at greater perturbation magnitudes. In addition, older adults did not demonstrate the ability to modulate maximal arm elevation velocity to match moderate perturbation intensity. This study builds upon a previously demonstrated lack of modulation of the stepping and trunk responses in older adults and is the first to show a lack of modulation in arm responses to slip-like perturbations in older adults.

### 4.1. Age-Related Impairment in the Modulation of Arm Responses

The ability to modulate balance responses to various perturbation intensities arises from both peripheral sensorimotor and central nervous system (CNS) mechanisms. As perturbation intensity increases, the demands on each of the balance systems also increases [20]. As a result, our findings must be interpreted in terms of both an impaired ability to perceive the intensity of a balance perturbation through peripheral and central sensory mechanisms and an impaired ability to generate higher velocities of arm movement through compromised neuromotor mechanisms.

We found that there was no difference in the maximal arm elevation displacement between older and younger adults. However, the velocity of maximal arm elevation was not modulated in response to the two intensities (1 and 2) in older adults, whereas the maximal arm velocity was progressively increased from intensity 1 to 2 to 3 in younger adults. Moreover, age-related differences in arm elevation velocity were identified at intensity 3 even although onset latencies were similar between younger and older adults. Over-all, these findings suggest that older adults are capable of moving the arms within an appropriate range of motion to restore CoM to a stable position without a delay in onset of the movement. However, the speed generating mechanisms appear to be impaired. These findings are consistent with the known declines in sensorimotor processing and neuromuscular control occurring with age. For example, motor and sensory events (connected information for performing voluntary movement) that occur within a certain temporal window are bound together to determine accurate judgements about changing external environment. The binding ability of sensory and motor input is compromised with age, resulting in poor spatial sensorimotor adaptations during movement response [21]. In addition, reduction in conduction velocity of peripheral afferent and efferent nerves [22], decreased presynaptic modulation of Ia-afferent input to motoneurons for force and postural control [23], and greater requirement of corticospinal excitability to control the soleus muscle activity for maintaining upright posture [24], all likely contribute to the impaired modulation ability of balance perturbation responses. Moreover, previous findings of reduced modulation of lower extremity stepping responses to different slip perturbations in older adults [16,25] suggest systemic underlying causes rather than age-related impairment affecting arm movement alone. 

One possible mechanism underlying our results are the age-related impairments in muscle quality and force-generating capacity. For example, a decreased proportion of type II fibers, increased connective tissue, fatty infiltration, and altered muscle metabolism have all been identified in older adults and each contributes to slowing of muscle contractile properties [26,27,28]. In addition, greater motor output variability caused by reduced synaptic input, fewer motor units, and less stable neuromuscular junctions can contribute to poor reactive performance in older adults [29]. These aging changes may partially ex-plain the inability for older adults to generate higher velocities of arm responses at relatively greater (moderate) perturbation intensity in the current study.

Age-related declines in peripheral and central sensory function may have also contributed to our results. Although the detection of a perturbation onset was not affected by age, impairments in the specificity of sensory feedback regarding the characteristics of the perturbation may account for the lack of modulation in older adults. Although there was no difference in arm elevation displacement between age groups, the change in arm elevation velocity in older adults was only apparent when the highest perturbation intensity occurs, suggesting that the sensitivity to more subtle changes in intensity (i.e., from intensity 1 to 2) may have been impaired in older adults. Age-related differences in the timing and accuracy in the detection of ankle movement direction and velocity have been identified previously [30,31]. Thus, delayed peripheral sensory transmission and central processing may have contributed to the mismatch between arm response velocity and relatively a bit higher perturbation magnitude. In addition, compared to younger adults, there was a tendency for older adults to move the hip joint along with the shoulder joints in response to unexpected A-P surface perturbations. This increased rigidity of upper body de-creases number of active degree of freedom for arm response control during balance recovery [32]. Age-related changes in plantar sensitivity [33] may also have led to further challenges in modulating arm responses, reducing the efficiency of interlimb coordination be-tween arms and legs for balance recovery [34].

### 4.2. Correlation between Arm Responses and Compensatory Stepping Stability

We also identified a positive correlation between maximal arm elevation velocity and first compensatory step stability at TD at the highest perturbation magnitude (level 3). Considering that lower extremity muscles can provide immediate joint torque to regain balance following external surface perturbations [35], participants in our study could have achieved balance recovery by mainly relying on stepping responses at the low and moderate perturbation magnitudes (level 1 and 2) to counteract the perturbation. When the perturbation intensity is more challenging, however, the associated increased postural instability requires both compensatory stepping and arm movements to recover balance completely [36,37,38]. Rapid arm responses to unpredictable slip perturbations serve to decelerate the backward movement of CoM and help to maintain the CoM within the initial BoS area, while a compensatory stepping serves to widen the BoS for balance recovery [39]. Our findings demonstrated that the stability of compensatory stepping is related to greater velocity of arm movement at the highest perturbation magnitude.

In both older and younger adults, the earlier onset of arm elevation with respect to the first compensatory foot LO provides further support for the importance of the arm response for balance recovery following slip-like perturbations. As others have reported, arm elevation acts as a counterweight to prevent the rapid movement of body’s CoM to-wards the direction of perturbation [40]. Arm responses also affect control of whole body angular momentum to help regain balance. For example, the rapid forward arm elevation during arm rotation in response to unpredictable slip perturbations generates a counter-rotation effect to offset the backward whole body angular momentum caused by unexpected anterior surface translation. This rapid arm response has been described as “inertial paddles” that decelerate and/or neutralize the backward angular momentum that may lead to backward falling [12]. 

### 4.3. Clinical Significance

Arm elevation responses demonstrate an important relationship with stability of compensatory stepping following slip-like perturbations [8]. Therefore, our findings of age-related decreases in arm response modulation ability, a mechanism to generate arm responses that match perturbation intensity, suggest that balance recovery may be delayed or ineffective in older adults and/or patients with neurological impairments. During actual falls, balance recovery responses that are unable to counteract a balance perturbation will lead to instability and falls. Results of this study suggest that fall prevention interventions may benefit from an emphasis on the speed of arm responses to unpredictable external balance perturbations at a range of intensities. Since balance response from unexpected perturbations is a flexible and functional motor skill including stretch reflexes for postural control, the interactions of multiple systems are organized to meet functional goals [41]. Thus, therapeutic intervention focusing on controlling arm response could be designed for people with neurological impairments and/or frail older adults to practice effective control of the body’s CoM during balance response. This intervention could improve centrally programmed movement synergies to adapt to various changing tasks and environmental demands required during balance recovery.

### 4.4. Limitations

There are a few study limitations that should be mentioned. First, given our small sample, size results must be interpreted with caution and future research is warranted. In addition, age-related changes, including muscle weakness, loss of ankle reflexes, and impaired sensation [42,43] may have confounded our findings. Despite of these limitations, this pilot study demonstrated age-related deficits in arm response modulation and a positive correlation between arm response velocity and compensatory stepping stability during balance recovery following slip perturbations.

## Data Availability

The data presented in this study are available on request from the corresponding authors.
